# Timely initiation of complementary feeding to children aged 6–23 months in rural Soro district of Southwest Ethiopia: a cross-sectional study

**DOI:** 10.1186/s12887-018-0989-y

**Published:** 2018-01-31

**Authors:** Bereket Yohannes, Elias Ejamo, Thilagavathi Thangavel, Mulugeta Yohannis

**Affiliations:** 1 School of public Health, Wolaita Sodo University, P.o.box 126 Wolaita Sodo, Ethiopia; 2 Centre for international Health, the University of Bergen, Bergen, Norway; 3 School of Public Health, Hawassa University, Hawassa, Ethiopia; 4 Soro district Health office, Hosanna, Ethiopia; 5 Damot Pulasa district, Wolaita Sodo, Ethiopia

**Keywords:** Timely initiation, Complementary feeding, Ethiopia

## Abstract

**Background:**

Poor complementary feeding practice to infants is one of risk factors for child undernutrition in Ethiopia. This would vary across the culturally and socioeconomically diverse settings in this country. Thus, this study was aimed to determine the proportion of timely initiated complementary feeding practice of women to their children aged 6–23 months in rural Soro district in Southwest Ethiopia.

**Methods:**

A community based crossectional survey was conducted in Soro district from August to September in 2015. Randomly selected 543 women having children aged 6–23 months were the final sampling units for this study. First, local administrative units (kebeles) of residents were randomly selected from such lists in the district. Secondly, the sample size was proportionally allocated to each selected kebele by population sizes. Individual households were selected by systematic random technique. Data was collected by using a structured questionnaire through face to face interview. Descriptive statistics was done for univariate results, and we applied bivariate logistic regression to look for crude association, and multivariate logistic regression to model predictors with effect measures and 95% confidence intervals (CI). Statistical significance was decaled at *P* < 0.05.

**Results:**

The proportion of timely initiated complementary feeding was 34.3% at 95%CI: (30.31, 38.29) in this study. Secondary and above education levels of respondents (AOR = 2.25 95%CI: 1.17, 4.30) and husbands (AOR = 2.33 at 95% CI: 1.06, 5.14), and maternal Postnatal Care visits (AOR = 1.94 at 95% CI: 1.19, 3.16) were found independent predictors for timely initiated complementary feeding practice in this study.

**Conclusions:**

Timely complementary feeding practice in the study area was low compared to the standard recommends for it. Education in general and equipping child bearing women with specific messages on Infant and Child Feeding Practices may improve infant and child feeding practice in the area. Optimizing utilization of Postnatal Care by post partum women and including specific advices on complementary feeding are recommended.

## Background

Malnutrition is responsible for about two third of all deaths among children under age 5 years. In Ethiopia, over a third of this is associated with inappropriate infant and young child feeding practice [1, 2] which includes suboptimal complementary feeding practice to infants. Complementary feeding means introducing an infant, other foods and liquids when breast milk alone is no longer sufficient [3–5]. After the first 6 months, breast milk alone is no longer adequate to meet the nutritional needs of the infants, therefore timely initiation of complementary foods to child’s diet is recommended [6–9]. This means introducing an infant with solid, semi-solid or soft foods at age 6 months. It is optimal or timely if at least initiated for infants aged 6–8 months [6, 7]. Appropriate complementary feeding should include a variety of foods to ensure nutritional requirements [6–9].

Estimates indicate that proper feeding of breast milk alone in the first six months could avert about 15% of infant deaths, and optimal complementary feeding could further reduce 6% of all deaths among children under 5 [10]. Nonetheless, in low income settings, where almost a fifth of all under age 5 dies due to causes that could be averted by proper complementary feeding [2]; suboptimal child feeding remains a risk factor for child undernutrition [8, 9, 11].

Despite its tremendous trend of improvement on some child nutrition indicators, Ethiopia still remains one of the high burden countries in child undernutrition [8, 12]. Only about half of infants in Ethiopia are timely initiated for complementary foods [2, 10, 12, 13]. It is much lower in Southern Ethiopia [8, 12].

Although several surveys documented on Infant and Young Child Feeding (IYCF) practices in Ethiopia, evidence is still limited on timely initiated complementary feeding practice and its specific predictors in its culturally and socioeconomically diverse settings like the Soro district in Southwest Ethiopia. Thus this study was aimed to determine the proportion of timely initiated complementary feeding practice of women to their infants in rural Soro district. The findings of this study might inform programs targeting infants and young children in the area and other similar settings. They are also expected to add on existing knowledge in the area.

## Methods

### Study design and setting

This study was aimed to determine the proportion of timely initiated complementary feeding practice of women to their infants in rural Soro district. It was a community based crossectional study among mothers who had children aged 6–23 months. The study was carried out in rural community of Soro district in South Ethiopia from August to September in 2015. The total census projected population in the district was estimated to be 239,058 which compose 15.6% children under age 5 years, 8.3% under age 3, and 3.5% infants and young children (6–23 months). About 55,700(23.3%) women in reproductive age group were expected to live in the district [14].

### Population and sampling

A sample size of 543 mothers was computed by using OpenEPi software with the assumptions for the design. The assumptions were 31% anticipated proportion of the outcome [13], 95% confidence level, 5% marginal error, and 6% for none-response [15]. First, out of the 46 rural residential kebeles (administrative units in the area) in the district 14 were selected by simple random sampling. Then sample size of study participant was allocated for each of the 14 units by proportion to population size. Then lists of mothers who had children 6–23 months of age in the each selected kebeles were obtained health posts in each kebele. In rural health posts in the area there family demographic and health profile registration folders (Family Folders). Thus we registered eligible mothers from these folders. Finally the study eligible mothers to child pairs were selected from each kebele by systematic random sampling technique.

### Data collection

Data was collected by face-to-face interview to mothers (caregivers) who had children aged 6–23 months through home to home visits. The questionnaire was adapted from WHO standard questions and indicators for assessing IYCF practices to suit the study setting [1, 16].

We used a structured questionnaire which was originally prepared in English and then translated to local Hadiya language and back to English by two people to keep consistency. The data collection tool was pretested to 27 mothers in villages which were not included in the study to validate content and language. Data was collected by fourteen nurses under supervision of 2 degree level trained health officers. The field team was trained for two on modules of the tool and field methods. The modules include; the study objective, relevance of the study, contents of the questionnaire, and ethics.

### Study variables

#### Outcome variable

Timely initiated complementary feeding.

### Exposure variables (covariates)

Sociodemographic factors: Age, sex, marital status, respondents’ educational status, educational status of husbands, occupation of the mother, family size.

Infant’s demographic related factors: age and sex of the child.

Household socioeconomic factors: house hold income.

Maternal health related characteristics: ANC follow up, child birth order, birth preparedness, type of delivery assistance and place of birth.

### Operational definitions

#### Timely initiation of complementary feeding

Introduction solid, semi-solid or soft foods to children between 6 and 8 months of age [6, 7].

#### Untimely initiation of complementary feeding

Introduction of solid, semi-solid or soft foods to infants before 6 months or beyond 8 months [6, 7].

### Statistical analysis

Data were checked for completeness and inconsistencies in the field on spot and corrections made. Then data were, entered in EPI info version 3.5.4 statistical software and exported to SPSS. It was cleaned and analysed by using SPSS version 16. Descriptive statistics was carried out for univariate results. Measures of central tendency and dispersion were used for describing the data. Binary logistic regression used to look for crude association between exposure and outcome variables. Exposure variables with *P* < 0.25 were considered as candidates for multivariate logistic regressions to control for confounding. Thus we reported Adjusted Odds Ratio (AOR) along with 95% CI for the effect measure for association, and statistical significance was declared at *P*-value < 0.05.

## Results

### Sociodemographic and socioeconomic characteristics

A total of 543 respondents having children aged 6–23 months were interviewed. Among the respondents 539(99.3%) were biological mothers and only 4 (0.7%) were other caregivers. The median (IQR) age of mothers was 28 years (26, 30). The majority of the respondents were Protestant 502(92.4%), and married 537 (98.9%). The median (IQR) age of the child age was 8(9, 17) in months and more than half of the children were male 283 (52.1%). Mothers’ who attended formal education was 243 (44.7%) and 330 (60.7%) husbands also attended school. The majority of mothers, 409 (75.3%), were housewives and husbands of 404 (75%) mothers were farmers by occupation. The median family size of the study households was six in number. Of the total households about 84.6% earn an average family monthly income ≤ 1000 Ethiopian Birr. Among the households 434 (79.9%) obtained food from farming followed by small scale business 73(13.4%) and formal employment 12(2.2%). Only 3 (0.6%) households had television and 152 (28%) had radio. It was found that, 22(4.1%) mothers read newspaper, 59(10.9%) mothers listened radio and 5(0.9%) mothers watched Television at least once a week (Table 1).

**Table 1:** Socio-demographic and socio economic related variables of mothers who had Children of 6–23 months age in rural community of Soro district, South Ethiopia, August, 2015 to September, 2015

Variable *n* = 543	Categories	Frequency	Percentage
Mothers’ age	15–24	76	14.0
	25–34	402	74.0
	35–40	65	12.0
Marital status	Married	537	98.9
	Unmarried	2	0.4
	Separated/Divorced	3	0.6
	Widowed	1	0.2
Religion	Protestant	502	92.4
	Orthodox	9	1.7
	Catholic	30	5.5
	Muslim	2	0.4
Ethnicity	Hadiya	526	96.9
	Kambata	11	2.0
	Amhara	3	0.6
	Gurage	1	0.2
	Others	2	0.4
Mothers’ educational status	No education	254	46.8
	Read and write	46	8.5
	Primary	186	34.3
	Secondary	52	9.6
	Tertiary	5	0.9
Mothers’ occupation	House wife	409	75.3
	Farmer	48	8.8
	Government employer	5	0.9
	Small business	65	12.0
	Causal work	16	2.9
Husbands’ Occupation(*n* = 539)	Not employed	20	3.7
	Farmer	404	75
	Employed	19	3.5
	Small scale trading	81	15
	Casual labour	11	2.0
	Daily labour	4	0.8
Monthly income	<=500	299	55.1
	501–1000	160	29.5
	1001–1500	25	4.6
	1501–2000	10	1.8
	2001 and above	9	1.7
	Don’t know	40	7.4
Source of monthly income	Farming	434	79.9
	Formal employment	12	2.2
	Casual labour	24	4.4
	Small scale business	73	13.4
Head of household	Husband	522	96.1
	Wife	16	2.9
	Oldest brother/sister	4	0.7
	Others	1	0.2

### Maternal health related characteristics

Among the respondents, 445 (82%), mothers had antenatal care follow up at least once during the last pregnancy. Only 134(30.2%) of mothers had at least 4 visits. Almost all of those who had ANC follow up 423 (95.1%) made the first their visit for the service (ANC 1) after second trimester or later. About 439(80.8%) mothers gave their recent child birth at home. Only106 (19.5%) mothers had received postnatal care (PNC) and 298 (54.9%) had complementary feeding counselling during recent pregnancy (Table 2)

**Table 2:** Obstetrics and maternal health service utilization related variables of mothers who had children 6–23 months of age in rural community of Soro district, South Ethiopia, August, 2015 to September, 2015

Variable	Categories	Frequency	Percentage
ANC follow up	Yes	445	82.0
	No	98	18
Number of ANC visit(*N* = 445)	Only once	53	11.9
	2–3 times	258	57.9
	>or = 4 times	134	30.2
Place of delivery	Home delivery	439	80.8
	Hospital	17	3.1
	Health center	68	12.52
	Health post	17	3.1
	Private clinic	2	0.4
Individual who assisted the delivery	Skilled Provider(Nurse/midwife/HO/Doctor)	94	17.3
	HEWs	17	3.1
	Non professionals(TBA and other persons)	432	79.6
Postnatal care	Yes	106	19.5
	No	437	80.5
Months of pregnancy during Initiation of ANC (n = 445	1–3	22	4.9
	4+	423	95.1
Counselling on complementary feeding	Yes	298	54.9
	No	237	43.6
	Don’t know	8	1.5

.

### Breast feeding practice

Nearly all children541 (99.6%) in this study had ever been breastfed and more than half 301(55.4%) were been initiated to breastfeed timely that is within one hour of birth. Some 501(92.1%) children were continued with breastfeeding at the period of study. Some 42 mothers (7.9%) stopped breast feeding before the child reaches 23 month of age.

### Complementary feeding practice

Majority of mothers 541 (99.6%) ever practiced breastfeeding. About 9 in 10 of all mothers 485(89.3%) who had breastfeeding experience had started complementary feeding at the time of the interview. Out of which 48(9.9%) were initiated before six month, 336(69.3%) at six and 101(20.8%) after six month of age. Among the respondents 410(75.5%) had introduced solid, semi solid or soft foods as complementary feeding at the time of the interview. Some 353 reported (65.0%) initiating complementary feeding after the infant’s eight months of age.

The result of this study shows the proportion of timely initiated complementary feeding in the study area was 34.3% 95% CI (30.31, 38.29) (Table 3).

**Table 3:** Child feeding practices of mothers who had children 6–23 months of age in rural community of Soro district, South Ethiopia, August to September, 2015

Variable	Categories	Frequency	Percentage
Ever breastfed	Yes	541	99.6
	No	2	0.4
Initiation of breast feeding	Immediately within 1st hour	301	55.4
	After 1st hour	234	43.1
	Don’t remember	8	1.5
Prelacteal feeding	Yes	18	3.3
	No	525	96.7
Ever started any CF	Yes	485	89.3
	No	58	10.7
Ever started solid,semi solid or soft foods	Yes	410	75.5
	No	133	24.5
Initiation of liquid foods(cow milk gruel)	Early	48	8.8
	Timely	419	77.2
	Lately	76	14
Initiation of solid, semi solid or soft foods	Early	4	0.7
	Timely	186	34.3
	Lately	353	65

### Factors associated with timely initiation of complementary feeding

Candidate variables for multivariate technique were identified by using bivariate analysis. The criteria was set to *P* < 0.25 as a yardstick. Accordingly; sociodemographic characteristics such as respondents’ achieved educational status, husbands’ educational status, and maternal age and maternal health service related characteristics such as Postnatal Care follow up, counselling during recent pregnancy, place of delivery and attendant were identified candidate variables for multivariate technique. We applied multivariate logistic regression for controlling possible confounders.

The findings of this study show mothers who had secondary school and above education were 2 folds more likely to timely initiate complementary feeding to their infants compared to those who did not attend formal education(AOR = 2.25 at 95% CI: 1.17, 4.30). Mothers living with their husbands trained to the level ability to read and write had also above 2 folds greater odds of timely initiating complementary foods to their infants compared to those who did not have formal education and were not able to read and write(AOR = 2.33 at 95%CI: 1.06, 5.14). On the other hand those mothers who had postnatal care were about 2 times more likely to timely initiate complementing food to their infants(AOR = 1.94 at 95%CI: 1.19, 3.16) compared to those who did not use the service (Table 4).

**Table 4:** Factors associated with Timely initiation of complementary feeding among mothers who had 6–23 months children in Soro district, South Ethiopia, Aug 2015 to Sept, 2015

Variables	Category	Complementary feeding timely Yes No		COR [95% C.I	AOR [95% C.I]
Mothers’ age	15–24	27	49	1.83(0.87, 3.86)	1.87(0.85,4.11)
	25–34	144	258	1.86(1.01,3.43)*	1.85(0.96,3.47)
	35–40	15	50	1	
Mothers’ educational status	No education	78	176	1	
	Read and write	19	27	1.58(0.83,3.02)	1.15(0.57,2.32)
	Primary	57	129	0.99(0.66,1.50)	0.81(0.51,1.29)
	Secondary and above	32	25	2.88(1.60,5.19)***	2.25(1.17,4.30)**
Educational status of husband(*n* = 539	No education	48	125	1	
	Read and write	17	19	2.33(1.11,4.85)*	2.33(1.06,5.14)*
	Primary	83	146	1.48(0.96,2.27)	1.35(0.84,2.16)
	Secondary and above	38	63	1.57(0.93,264)	1.20(0.67,2.18)
PNC follow up	Yes	53	53	2.28(1.48,3.52)***	1.94(1.19,3.16)**
	No	133	304	1	
Place of delivery	Home delivery	140	299	1	
	Institutional delivery	46	58	1.69(1.10,2.62)*	1.40(0.36,5.38)
Assistant of the delivery	Non professional	138	294	1	
	Health professional	48	63	1.62(1.06,2.48)*	0.79(0.21,2.99)
Counselling on complementary feeding	Yes	118	180	1.62(1.18,2.45)**	1.48(1.00,2.19)
	No	68	170	1	

Foot note * = *p* < 0.05 ** = *p* < 0.01 *** = *p* < 0.001

## Discussion

The proportion of timely initiated complementary feeding in study was 34.3%. Parental education and maternal postnatal care follow-up were independent predictors for the outcome. The timely initiated complementary feeding practice in this study was much lower than findings from India(55%), Nepal (70%), Bangladesh (71%), Sirlanka (84%), and Kenya (80.6%) [17–19]. The observed disparity might be due to cultural barriers, and socioeconomic and health care access disparities.

Much lower proportion of infants in Soro were timely initiated to complementary feeding in compared the national figure for Ethiopia in 2011(51%) [12] and in 2016(56%) [8]. Similarly it was also lower than data from Tigray (48.4%) in Northern Ethiopia [13], Sidama (72.2%) [20] in the South. This might be due to sociodemographic differences including the low level of parental education in our settings. It might also be explained by the low coverage and utilization of maternal health services; particularly poor postnatal care utilization in Soro coupled missed opportunities during visits for maternal services. On the other hand, timely initiated complementary feeding practice in this study is in line with ‘alive thrive survey’ report for Southern Ethiopia in 2011 (31.2%) [13]. This might imply that timely initiation of complementary feeding would be low in Southern Ethiopia’s across the different settings and the disparity might also vary with the diversity and socioeconomic variations.

This study indicated that mothers educated to secondary and above levels were above two folds more likely to timely initiate complementary feeding to infants compared to those who did not attend any school level. Similar findings were reported from Mekelle town in North Ethiopia, Nairobi in Kenya [21, 22], and Nepal [17]. The better educated mothers would have good knowledge about the importance of complementary feeding practice; they might also better understand the message. They might have better connection to nutrition information sources.

Paternal education also enhances mothers to timely initiate complementary feeding by more than 2 folds compared to who had husband with no formal education. Similar finding was reported from Axum town in North Ethiopia [23]. This might be argued that education might enable the husbands to better understand their wives and provide help by approving what mothers would like to do to keep their children healthy. The educated husbands might enhance wives awareness on to timely initiation of complementary feeding to their infant.

Postnatal care user mothers were almost 2 times more likely to timely initiate complementary feeding to their infants compared to those who did not follow the service. Similar finding was reported from Kamba district in Southwest Ethiopia [15]. Mothers who had no postnatal care follow up would start complementary feeding earlier (before 6 months) or later (after 8 months) compared to mothers who followed the care. A postnatal period could be an ideal time to counsel mothers on optimal complementary feeding practice [21, 23, 24].

Some level of recall bias was expected while interviewing respondents particularly none-mother ones in this study. The study was also limited in scope for exploring socio-cultural and behavioural factors. Using WHO guidelines and indicators might be strength in this study.

## Conclusions

Only about a third of mothers in this study timely initiated complementary feeding to their infants, which was much lower than the national figure for Ethiopia and also far lower than the WHO recommendations. Higher (secondary and above) parental educational status, and better postnatal care utilization were associated with timely initiation of complementary feeding. Maternal education to higher might have enabled them to comprehend packages of routine messages pertaining to complementary feeding. Husbands’ education was also equally important for timely complementary feeding to their infants. Authors recommend health workers should give special emphasis to mothers with low literacy while giving services. Postnatal care utilization should be strengthened on one hand and it should get due emphasis for counselling mothers on appropriate optimal complementary feeding practices. Further studies are recommended to evaluate the association between maternal prenatal (ANC) and delivery service utilization with appropriate complementary feeding practice. Further evidence is also needed on its socio-cultural and behavioural barriers in the area and beyond.

## Abbreviations

ANC: Antenatal care
AOR: Adjusted Odds Ratio
BF: Breast Feeding
CF: Complementary Food
CI: Confidence Interval
EDHS: Ethiopian Demographic Health Survey
FMOE: Federal Ministry of Education
HEW: Health Extension Worker
HO: Health Officer
IYCF: Infant and Young Child Feeding
NORHED: Norwegian Programme for Capacity Development in Higher Education and Research for Development
PNC: Postnatal Care
PPS: Proportional to Population Size
SENUPH: South Ethiopia Network Universities in Public health
SNNPR: Southern Nation Nationality Peoples Region
SPSS: Statistical Package for Social Sciences
TBA: Traditional Birth Attendant
WHO: World Health Organization

## References

[1] Organization, W.H. and Unicef, Global strategy for infant and young child feeding*.* 2003.

[2] UNICEF., Infant and young child feeding, nutrition section program. 2011, UNICEF:. New York.

[3] Lutter C (2003). Meeting the challenge to improve complementary feeding. SCN news.

[4] Organization, W.H. Strengthening action to improve feeding of infants and young children 6–23 months of age in nutrition and child health programmes. in Report of proceedings. 2008.

[5] Organization, P.A.H., Guiding principles for complementary feeding of the breastfed child. 2003, Washington, DC: Pan American Health Organization.

[6] Daelmans B, Dewey K, Arimond M (2009). New and updated indicators for assessing infant and young child feeding. Food & Nutrition Bulletin.

[7] Organization, W.H., Guiding principles for complementary feeding of the breastfed child. 2001, World Health Organization: Geneva: .

[8] [Ethiopia], C.S.A.C. and ICF, Ethiopia Demographic and Health Survey 2016, in Final report. 2017, Central statistical authority: Addis Ababa Ethiopia, Rockville, Maryland, USA.

[9] Institute, I.F.P.R. Global Nutrition Report 2016, in From promise to impact 2016, international food policy research institute: Washington. DC.

[10] Lutter CK (2011). Undernutrition, poor feeding practices, and low coverage of key nutrition interventions. Pediatrics.

[11] Kumar D (2006). Influence of infant-feeding practices on nutritional status of under-five children. The Indian Journal of Pediatrics.

[12] Macro, C.S.A.E.a.O., Ethiopia Demographic and Health Survey (2011). 2011, central statistical agency and ORC macro: Addis Ababa.

[13] Ali D (2011). Alive and thrive baseline survey report: Ethiopia.

[14] office, S.w.H., Soro woreda Health sector planning,monitoring and evalution core process. 2014.

[15] Agedew, E., et al., Early Initiation of Complementary Feeding and Associated Factors among 6 Months to 2 Years Young Children. Kamba Woreda, South West Ethiopia: A Community–Based Cross-Sectional Study. J Nutr Food Sci, 2014. 4(314): p. 2.

[16] Organization, W.H., Indicators for assessing infant and young child feeding practices: part 2: Measurement 2010.

[17] Joshi N (2012). Determinants of inappropriate complementary feeding practices in young children in Nepal: secondary data analysis of demographic and health survey 2006. Maternal & child nutrition.

[18] Senarath U (2012). Determinants of inappropriate complementary feeding practices in young children in Sri Lanka: secondary data analysis of demographic and health survey 2006–2007. Maternal & child nutrition.

[19] Ogunlesi T (2015). Determinants of timely initiation of complementary feeding among children aged 6-24 months in Sagamu, Nigeria. Niger J Clin Pract.

[20] Tessema M, Belachew T, Ersino G. Feeding patterns and stunting during early childhood in rural communities of Sidama, South Ethiopia. Pan African Medical Journal. 2013:14(1). 10.11604/pamj.2013.14.75.1630 PMC3641921 23646211

[21] Shumey A, Demissie M, Berhane Y (2013). Timely initiation of complementary feeding and associated factors among children aged 6 to 12 months in northern Ethiopia: an institution-based cross-sectional study. BMC Public Health.

[22] Kimani-Murage EW (2011). Patterns and determinants of breastfeeding and complementary feeding practices in urban informal settlements. Nairobi Kenya BMC Public Health.

[23] Yemane S, Awoke T, Gebreslassie M (2014). Timely initiation of complementary feeding practice and associated factors among mothers of children aged from 6 to 24 months in Axum town, north Ethiopia. Sciences.

[24] Abera K. Infant and young child feeding practices among mothers living in Harar, Ethiopia. Harar bulletin of Health Sciences. 2012;4

